# Procalcitonin as marker for bacterial coinfection among adult COVID-19 patients in a tertiary-care hospital in the Philippines

**DOI:** 10.1017/ash.2022.73

**Published:** 2022-05-16

**Authors:** Angelie Lorraine Pesebre, Cybele Lara Abad, Jan Jorge Francisco, Justin Allister Ong

## Abstract

**Background:** Antimicrobials are often given to patients with COVID-19 despite the absence of a bacterial coinfection. Procalcitonin (PCT), when elevated, often indicates the presence of a bacterial infection and is used to guide empiric antibiotic therapy. We sought to determine the utility of PCT and the optimal cutoff value of PCT among patients with COVID-19. **Methods:** We retrospectively reviewed all COVID-19 confirmed ca-ses hospitalized in our institution from March to December 2020. Of 729 cases, we included 403 (55.3%) who had baseline PCT and blood or respiratory tract specimens (eg, sputum, endotracheal aspirate) within 48 hours of admission. Participants were classified according to PCT levels and COVID-19 severity. A receiver operating characteristic (ROC) curve analysis was performed. The area under the curve (AUC) obtained was used to compute the possible optimal cutoff value using the Youden index. A χ^2^ test was used to define association between groups according to the characteristics of variables. **Results:** Of a total cohort of 403, 245 (57%) were male, with an overall median age of 60 years (range, 22–94). Overall, 28 presented with mild COVID-19, 194 presented with moderate COVID-19, and 181 presented with severe or critical COVID-19. Moreover, 363 (90%) were given antibiotics. Of 28 with mild COVID-19, 22 (79%) received empiric antibiotics. The rate of bacterial coinfection was high at 28% (113 of 403). *Klebsiella pneumoniae* was the most commonly identified microorganism: 52 (19.5%) of 266 patients. Based on the ROC curve, the optimal cutoff for PCT was 4.72 ng/mL, with 97% specificity and only 6% sensitivity. Only 17 participants had PCT > 4.72 ng/mL. Of these, 1 was mild, 5 were moderate COVID-19, 8 had severe COVID-19, and 3 had critical COVID-19; all received antibiotic therapy. **Conclusions:** In our cohort, the rate of bacterial coinfection was high. A PCT of >4.72 ng/mL increased the likelihood of a coinfection. However, PCT had poor sensitivity and may not detect the presence of bacterial coinfection, especially when used alone. Serial PCT monitoring, its use in conjunction with other markers, or as a prognostic tool, need to be explored further.

**Funding:** None

**Disclosures:** None

